# Multi-year whole-blood transcriptome data for the study of onset and progression of Parkinson’s Disease

**DOI:** 10.1038/s41597-019-0022-9

**Published:** 2019-04-05

**Authors:** Matthew N. Z. Valentine, Kosuke Hashimoto, Takeshi Fukuhara, Shinji Saiki, Kei-ichi Ishikawa, Nobutaka Hattori, Piero Carninci

**Affiliations:** 1 Division of Genomic Medicine, RIKEN Center for Integrative Medical Sciences, Yokohama, Japan; 2 Department of Neurology, Juntendo University School of Medicine, Tokyo, Japan

**Keywords:** RNA sequencing, Parkinson's disease, Biomarkers, Gene expression

## Abstract

Parkinson’s disease (PD) is an age-related, chronic and progressive neurodegenerative disorder characterized by a loss of multifocal neurons, resulting in both non-motor and motor symptoms. While several genetic and environmental contributory risk factors have been identified, more exact methods for diagnosing and assessing prognosis of PD have yet to be established. Here we describe the generation and validation of a dataset comprising whole-blood transcriptomes originally intended for use in detection of blood biomarkers and transcriptomic network changes indicative of PD. Whole-blood samples extracted from both early-stage PD patients and healthy controls were sequenced using no-amplification non-tagging cap analysis of gene expression (nAnT-iCAGE) to analyse differences in global RNA expression patterns across the conditions. Subsequent sampling of a subset of PD patients one-year later provides the opportunity to study changes in transcriptomes arising due to disease progression.

## Abstract (summary)

**Table:** 

Design Type(s)	disease state design • transcription profiling design • parallel group design
Measurement Type(s)	transcription profiling assay
Technology Type(s)	RNA sequencing
Factor Type(s)	age • duration of disease • biological sex • disease severity measurement • experimental condition
Sample Characteristic(s)	Homo sapiens • blood

Machine-accessible metadata file describing the reported data (ISA-Tab format)

## Background & Summary

Parkinson’s disease (PD) is the second most common neurodegenerative disorder with an average age of onset of 60 years and a prevalence of about 1–2% in industrialized countries^1^. The overall incidence of the disease is increasing, and projections indicate that there will be three times as many individuals affected by PD^2^ by 2030. PD is characterized by the loss of dopaminergic neurons of the substantia nigra^3^, as well as formation of intracellular Lewy bodies consisting primarily of α-synuclein^4^. The resulting depletion of dopamine (DA) manifests symptoms broadly relating to movement and coordination: resting tremors, bradykinesia, rigidity and postural instability^3^. Additional non-motor symptoms often precede the more overt motor features by several years, including anosmia, sleep disorders and constipation^5^. Most PD cases are classified as sporadic, with inherited familial forms of the disease accounting for a mere 5% of all cases^3^. Though the exact cause is unknown, a combination of genetic predisposition (including mutations in the leucine-rich repeat kinase 2 (LRRK2) gene^6,7^, α-synuclein^8^ (SNCA), parkin^9,10^ (PARK2), PTEN-induced putative kinase 1^11^ (PINK1) and DJ-1^12^ (PARK7)) and environmental factors are thought to be the primary events in disease induction.

With no definitive test for PD, current diagnosis is dependent on clinical observation of overt symptoms. However, overlap with other neuropathological disorders can make accurate diagnosis difficult, leading to misdiagnosis and incorrect treatment plans^13,14^. Additionally, the presentation of symptoms, especially in early PD, is highly heterogeneous in nature^15^, further muddying the waters with regards to confidence in individual diagnoses. There is a high demand for diagnostic procedures utilizing clinically-relevant biomarkers of PD: the ability to routinely test for biomarkers through a minimally invasive approach would make for a powerful diagnostic tool. This is especially true for biomarkers indicating the earliest stages of PD, as early diagnosis and intervention will likely lead to better prognostic outcomes, as well as limiting misdiagnoses.

In previous work, we showed how a series of acylcarnitine metabolites could be detected in the blood metabolome profiles of PD patients, serving as a biomarker of PD at its earliest stage^16^. The sensitive detection of these biomarkers by LC-MS/MS opens up the possibility of diagnosis from blood samples, even in the early stages of PD. Further to this, we decided to investigate whether yet more PD biomarkers could be found within the blood transcriptome. In fact, Infante *et al*. previously reported differences in transcriptomic expression between LRRK2 G2019S mutated patients and idiopathic PD patients by RNA-seq^17,18^, indicating the potential of such an analysis for highlighting differences between PD subtypes and between PD and healthy controls.

For this study, we collected whole blood samples from PD patients at an early stage of disease progression and healthy controls, with an aim to identify potent transcriptomic biomarkers at high resolution using an unbiased analysis method. Specifically, we utilized the no-amplification non-tagging cap analysis of gene expression (nAnT-iCAGE) protocol^19^ to capitalize on the strengths of CAGE-sequencing, namely the ability to determine the RNA expression level of both known and unknown transcripts and the transcription start site (TSS) utilized, as well as prediction of promoter regions^20^. CAGE-sequencing also limits potential bias-generating steps introduced in the sample preparation of other sequencing methodologies. With the nAnT-iCAGE sequencing protocol in particular there is no need for PCR amplification, which is commonly carried out prior to sequencing and requires post-sequencing computational cleanup to mitigate bias introduction. Further, nAnT-iCAGE avoids the poly-A based enrichment that was carried out in previous transcriptomic analyses of blood in Parkinson’s disease^17,18^. As a result, with this dataset it is possible to quantify and analyse important non-polyadenylated transcripts, such as bidirectionally transcribed enhancer RNAs^21^.

The samples collected and described in this paper include 39 PD and 20 control whole blood transcriptome samples^22^. These samples focus only on early stage PD, but encompass a range of ages and genders of participants, as well as differences in clinical scores (Table 1), and thus may account for some of the heterogeneity seen across PD patients. Additionally, the samples described here were collected over two years, thus allowing for some analysis of disease progression within early PD, in addition to highlighting differences between control and disease conditions.

**Table 1:** Metadata of all sequenced samples available through the NBDC human database.

Sample	Condition	Sequencing year	Gender	Age	H&Y	UPDRSIII	Disease duration (until study start)	LEDD	Age at onset	Y1–Y2 pair
CNhi10654.ACC	Y1.Ct	1	F	83	na	na	na	na	na	Unpaired
CNhi10654.CAC		1	M	54	na	na	na	na	na	Unpaired
CNhi10655.AGT		1	M	64	na	na	na	na	na	Unpaired
CNhi10655.GCG		1	F	73	na	na	na	na	na	Unpaired
CNhi10656.ATG		1	F	78	na	na	na	na	na	Unpaired
CNhi10656.TAC		1	M	62	na	na	na	na	na	Unpaired
CNhi10656.ACG		1	F	60	na	na	na	na	na	Unpaired
CNhi10657.ACC		1	F	64	na	na	na	na	na	Unpaired
CNhi10657.CAC		1	F	50	na	na	na	na	na	Unpaired
CNhi10657.GCT		1	M	67	na	na	na	na	na	Unpaired
CNhi10654.AGT	Y1.PD	1	F	67	1	2	1	75	66	Unpaired
CNhi10654.GCG		1	M	75	2	14	1	0	74	CNhi10846.GCT
CNhi10654.ATG		1	M	74	2	9	3	555	71	Unpaired
CNhi10654.TAC		1	F	64	2	13	1	130	63	CNhi10847.ACC
CNhi10654.ACG		1	M	49	2	44	2	500	47	CNhi10847.CAC
CNhi10654.GCT		1	F	60	1	4	1	67	59	CNhi10847.ATG
CNhi10655.ACC		1	F	67	2	14	1	438	66	CNhi10847.TAC
CNhi10655.CAC		1	M	44	1	1	1	50	43	CNhi10847.ACG
CNhi10655.ATG		1	F	62	1	14	2	375	60	Unpaired
CNhi10655.TAC		1	M	71	1	6	2	325	69	Unpaired
CNhi10655.ACG		1	F	73	1	4	1	475	72	CNhi10847.GCT
CNhi10655.GCT		1	F	75	1	3	1	300	74	CNhi10848.ACC
CNhi10656.ACC		1	M	61	1	4	3	375	58	CNhi10848.CAC
CNhi10656.CAC		1	F	60	2	36	3	650	57	Unpaired
CNhi10656.AGT		1	F	58	1	8	3	500	55	Unpaired
CNhi10656.GCG		1	F	50	2	2	1	130	49	Unpaired
CNhi10656.GCT		1	F	68	2	12	2	225	66	CNhi10848.AGT
CNhi10657.AGT		1	F	70	2	7	2	600	68	CNhi10848.GCG
CNhi10657.GCG		1	F	67	2	13	2	183	65	CNhi10848.GCT
CNhi10657.ATG		1	F	70	1	4	1	0	69	Unpaired
CNhi10657.TAC		1	F	76	1	10	1	150	75	Unpaired
CNhi10657.ACG		1	F	65	1	5	1	150	64	Unpaired
CNhi10846.AGT		2	F	73	1	4	4	150	69	CNhi10849.AGT
CNhi10846.GCG		2	M	45	2	1	2	392	43	CNhi10849.GCG
CNhi10846.ATG		2	M	61	1	6	4	600	57	CNhi10849.ATG
CNhi10846.TAC		2	M	47	1	5	3.5	0	44	CNhi10849.TAC
CNhi10846.ACG		2	M	62	2	14	4	362	58	CNhi10849.ACG
CNhi10846.ACC	Y2.Ct	2	F	79	na	na	na	na	na	Unpaired
CNhi10846.CAC		2	F	76	na	na	na	na	na	Unpaired
CNhi10847.AGT		2	M	72	na	na	na	na	na	Unpaired
CNhi10847.GCG		2	M	78	na	na	na	na	na	Unpaired
CNhi10848.ATG		2	M	75	na	na	na	na	na	Unpaired
CNhi10848.TAC		2	F	55	na	na	na	na	na	Unpaired
CNhi10848.ACG		2	F	73	na	na	na	na	na	Unpaired
CNhi10849.ACC		2	M	43	na	na	na	na	na	Unpaired
CNhi10849.CAC		2	F	53	na	na	na	na	na	Unpaired
CNhi10849.GCT		2	M	42	na	na	na	na	na	Unpaired
CNhi10846.GCT	Y2.PD	2	M	76	2	9	1	350	74	CNhi10654.GCG
CNhi10847.ACC		2	F	65	2	5	1	470	63	CNhi10654.TAC
CNhi10847.CAC		2	M	50	2	25	2	710	47	CNhi10654.ACG
CNhi10847.ATG		2	F	61	2	4	1	150	59	CNhi10654.GCT
CNhi10847.TAC		2	F	68	1	14	1	438	66	CNhi10655.ACC
CNhi10847.ACG		2	M	45	2	1	1	175	43	CNhi10655.CAC
CNhi10847.GCT		2	F	74	1	6	1	525	72	CNhi10655.ACG
CNhi10848.ACC		2	F	76	1	4	1	399	74	CNhi10655.GCT
CNhi10848.CAC		2	M	62	1	1	3	413	58	CNhi10656.ACC
CNhi10848.AGT		2	F	69	2	13	2	625	66	CNhi10656.GCT
CNhi10848.GCG		2	F	71	2	8	2	330	68	CNhi10657.AGT
CNhi10848.GCT		2	F	68	2	23	2	210	65	CNhi10657.GCG
CNhi10849.AGT		2	F	74	1	2	4	150	69	CNhi10846.AGT
CNhi10849.GCG		2	M	45	1	1	2	445	43	CNhi10846.GCG
CNhi10849.ATG		2	M	62	1	4	4	750	57	CNhi10846.ATG
CNhi10849.TAC		2	M	48	1	7	3.5	181	44	CNhi10846.TAC
CNhi10849.ACG		2	M	63	3	8	4	605	58	CNhi10846.ACG

## Methods

### Blood sample collection

**Fig. 1:**
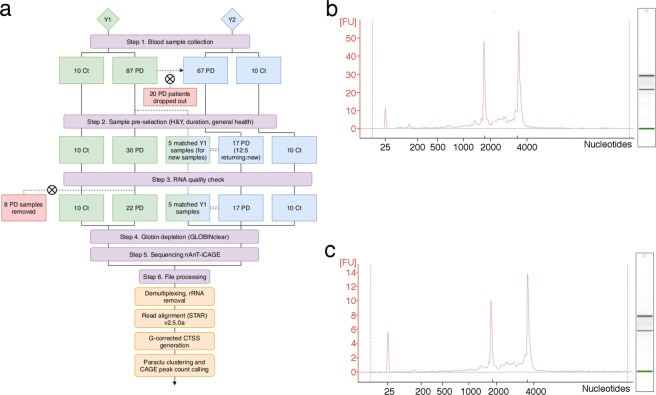
Study work flow from sample preparation through to sequence processing. (**a**) Flow chart showing the key stages of the study, and the number of participants going through to final sequencing. Pre-sequencing RNA quality control check used BioAnalyzer, and example results for (**b**) Ct and (**c**) PD samples show good quality RNA for library preparation.

PD was diagnosed according to the Movement Disorders Society Clinical Diagnostic Criteria for Parkinson’s disease^23^. Blood samples were collected from 87 PD and 10 control patients in the first year of the study (Y1) and from 67 PD (continuing from Y1) and 10 control patients in the second year (Y2; see Fig. 1a for flow diagram). All blood was collected and immediately stored at −80 °C in PAXgene Blood RNA tubes (PreAnalytiX). From the initial set of PD samples, 30 were pre-selected for RNA extraction (Fig. 1, step 2) on the basis of the following criteria: non-smokers, no significant previous disease, early stage of disease progression (one or two on the Hoehn & Yahr (H&Y)^24^ scale) and a duration since disease onset of 1–3 years. Going into Y2, 12 of the sequenced Y1 patients remained in the study. Five replacement patient samples were chosen for sequencing from the remaining pool of 67 samples collected in Y2. The initial criteria were relaxed to allow a duration since onset of up to four years, though these new samples were still required to be low on the H&Y scale. Both the stored blood samples from Y1 and the newly collected Y2 samples for these five patients were sequenced along with the other 12 Y2 samples. The use of human blood was approved by the ethics evaluation committee of Juntendo University (Approval Number: 15–104) and the ethics review committee of RIKEN (H26-27). Informed consent was obtained from each participant.

### CAGE library preparation

RNA was extracted from blood samples using the PAXgene Blood miRNA kit (PreAnalytiX). Following RNA extraction, samples with low quality scores (<6.5 RIN) or low concentrations of RNA (<4.5 μg) were removed (see *Technical Validation*), leaving 22 PD and 10 control samples in Y1, and 17 PD and 10 control samples in Y2 (Fig. 1a, step 3). It is well documented that the blood transcriptome is highly saturated by globin RNAs^25^ (predominantly alpha and beta haemoglobins), which has a masking effect on the remaining lower abundance transcripts. To limit this effect, samples remaining after the RNA isolation step were depleted of haemoglobins using the GLOBINclear^TM^ kit (Thermo Fisher Scientific). nAnT-iCAGE libraries were prepared following the protocol described in Murata *et al*.^19^. Briefly, 3 ng of total RNAs were used for the synthesis of cDNA with random primers. The cDNAs with an intact 5′-end were captured by streptavidin-coated magnetic beads, ligated to a 5′ linker containing a barcode sequence and further ligated to a 3′ linker. A second strand was synthesized to generate the final dsDNA product used for sequencing. CAGE libraries were sequenced with the 50 bp single-end mode on the Illumina HiSeq 2500 platform.

### Read alignment and processing steps

Raw sequencing files available from above^22^ require processing before data analysis, and what follows is a brief description of the steps involved. Multiplexed reads should be split by barcodes, and ribosomal RNAs removed using rRNAdust v1.06 (in-house scripts, see *Code availability*). General quality of the FastQ files can be assessed per sample using FastQC^26^ (see *Technical validation*). The extracted CAGE tags can then be aligned to the current human reference genome (hg38) using a number of aligners (here STAR^27^ version 2.5.0a was used; see *Technical validation*). A genome-wide transcription start site (TSS) map of single-nucleotide resolution can be generated from the 5′ coordinates of the CAGE tags, which can then be used to define distinct TSS peaks (for instance using Paraclu^28^). Note, the CAGE protocol is known to introduce an additional G nucleotide to the 5′ end of the CAGE tag, so a transformation algorithm must be used to correct for this systematic G-addition bias (see *Code availability*).

## Data Records

All raw nAnT-iCAGE sequencing data (FASTQ files, samples 1–64 corresponding to Y1 and Y2 data) as well as sample metadata are available through the NBDC human database^30^ (https://humandbs.biosciencedbc.jp/en/) under accession number JGAS00000000119 (controlled access)^22^.

## Technical Validation

### RNA quality control

Extracted RNA was analysed using the Agilent 2100 bioanalyzer, assessing quality and concentration of intact RNA to determine suitability for subsequent sequencing. Example high quality outputs for Y1 control (Fig. 1b) and PD (Fig. 1c) samples are shown. Only samples with a concentration in excess of 4.5 ug and RNA integrity number of 7 or higher were selected for CAGE sequencing.

### Read quality and accurate base-calling

**Fig. 2:**
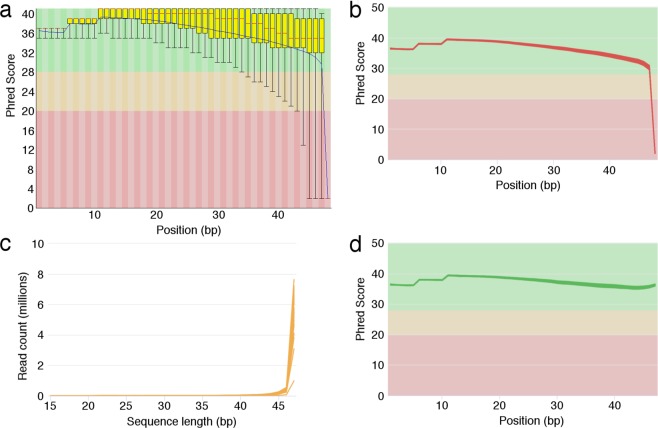
Post-sequencing quality control of FASTQ files using FastQC. (**a**) Example FastQC plot for a control sample showing a drop in per base quality scores towards the end of the 50 bp read length. (**b**) Aggregated FastQC plots reveal this is a widespread phenomenon affecting all of the samples. (**c**) Trimming sequenced reads based on quality score introduces variety in sequence length distribution, though the majority are still greater than 45 bp in length. (**d**) After trimming, all samples pass the mean quality score test in FastQC.

FastQC was used to assess the quality of the sequenced reads on a per sample basis, with a focus on the per base sequence quality. FastQC looks at the Phred quality score, calculated by comparing read signals to the probability of accurate base-reading. Phred scores are related to base-calling error probabilities in a logarithmic manner (Q = −10 log_10_ P), such that scores of 50, 40 and 30 indicate base call accuracies of 99.999%, 99.99% and 99.9% respectively. An example Y1 control sample is shown in Fig. 2a, with an aggregated plot of all Y1 samples generated using MultiQC^31^ shown in Fig. 2b. Though the Phred scores at the majority of the base positions were high, indicating high accuracy in the assigned base at the given nucleotides, the final base at position 48 had a very low average score of 2. Trimming the reads to exceed a mean Phred score of 30 can be easily carried out (for instance using the FASTQ Quality Trimmer from FASTX^32^), creating a set of sequences that are of high quality and unambiguous in nature. Trimming in this manner introduces variation in the sequence length, though the majority of reads are over 45 bases in length (Fig. 2c,d) indicating minimal loss from the original 48 base length.

### CAGE quality control

**Fig. 3:**
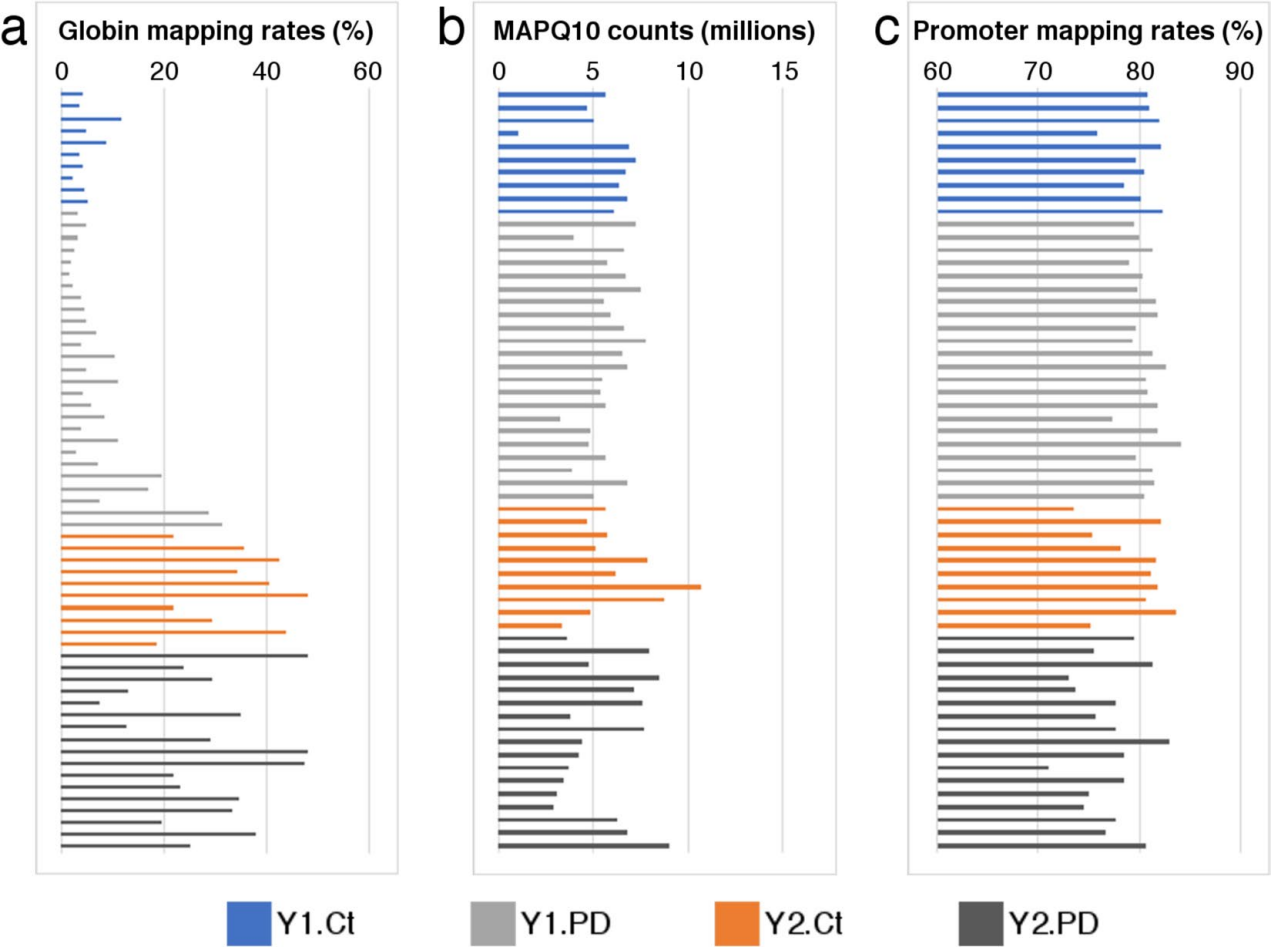
Mapping statistics and quality control of CAGE data. (**a**) Percentage of all sequenced CAGE tags (including multimapping tags) originating from haemoglobin genes. **(b**) Number of high quality, unambiguously mapping tags across all the samples. (**c**) Percentage of the MAPQ10 tags that overlap with the FANTOM5^33^ promoter regions.

The GLOBINclear^TM^ kit successfully depleted the Y1 samples of haemoglobins, with the remaining globin mRNAs accounting for around 5.1% of the total sequenced tags (Fig. 3a). The proportion of globin tags in the Y2 sequenced samples was higher, averaging 29.1% of total tag counts, indicating the globin depletion was not as efficient (Fig. 3a). Many of these tags cannot be unambiguously aligned to the genome (so-called multimappers), and thus can be easily removed before downstream analyses. In general, we obtained a high rate of CAGE tags mapping unambiguously to the hg38 human genome using STAR, with an average MAPQ10 count of 5.9 million tags across the two sequencing batches (Fig. 3b). Coupled with the depletion of globin RNAs, this indicates a high-quality set of sequencing samples that can be used for blood transcriptomic analysis of early PD. Furthermore, the samples show a high degree of consensus with FANTOM5 promoters, with an average of 77.8% of all tags overlapping promoter regions (Fig. 3c). One important caveat to make note of is that one of the Y1 control samples had a lower sequencing depth, with total MAPQ10 counts of less than 1 million (Fig. 3b). Despite the fact that this sample clusters separately from the remainder of the control samples, it is still highly similar. For instance, the number of detected promoters for this sample is only slightly reduced compared with the overall promoter mapping rate for control samples (75.85% of FANTOM5 promoters versus 79.79 ± 2.8%; Fig. 3c), showing that the majority of promoters expressed in other control samples are also expressed here.

## ISA-Tab metadata file

Download metadata file

## Data Availability

A number of in-house scripts are commonly used for the processing of the raw FASTQ files before alignment as well as for correction of the CAGE specific sequencing bias mentioned above (and described in more detail in supplementary note 3-e of Carninci *et al*.^20^). A brief description of these scripts follows: splitByBarcode is used to split multiplexed sequences into constituent sample FASTQ files and can be found in the MOIRAI system^29^; rRNAdust removes all sequences that match to known rRNA sequences with two or fewer errors and is freely available through the FANTOM5 website (http://fantom.gsc.riken.jp/5/suppl/rRNAdust/); starbam2gcorrectedctss is a shell script used to convert BAM files to CTSS bed files, correcting for any additional Gs at the 5′ end, and is available upon request.
